# Inhibition of TGFβ‐activated protein kinase 1 ameliorates myocardial ischaemia/reperfusion injury via endoplasmic reticulum stress suppression

**DOI:** 10.1111/jcmm.15340

**Published:** 2020-05-07

**Authors:** Jingjing Zeng, Qike Jin, Yongxue Ruan, Changzheng Sun, Guangyu Xu, Maoping Chu, Kangting Ji, Lianpin Wu, Lei Li

**Affiliations:** ^1^ Institute of Cardiovascular Development and Translational Medicine The Second Affiliated Hospital & Yuying Children’s Hospital of Wenzhou Medical University Wenzhou China

**Keywords:** endoplasmic reticulum stress, myocardial ischaemia/reperfusion, ROS, TAK1

## Abstract

Transforming growth factor β‐activated protein kinase 1 (TAK1) involves in various biological responses and is a key regulator of cell death. However, the role of TAK1 on acute myocardial ischaemia/reperfusion (MI/R) injury is unknown. We observed that TAK1 activation increased significantly after MI/R and hypoxia/reoxygenation (H/R), and we hypothesized that TAK1 has an important role in MI/R injury. Mice (TAK1 inhibiting by 5Z‐7‐oxozeaenol or silencing by AAV9 vector) were exposed to MI/R injury. Primary cardiomyocytes (TAK1 silencing by siRNA; and overexpressing TAK1 by adenovirus vector) were used to induce H/R injury model in vitro. Inhibition of TAK1 significantly decreased MI/R‐induced myocardial infarction area, reduced cell death and improved cardiac function. Mechanistically, TAK1 silencing suppressed MI/R‐induced myocardial oxidative stress and attenuated endoplasmic reticulum (ER) stress both in vitro and in vivo. In addition, the inhibition of ROS by NAC partially reversed the damage of TAK1 in vitro. Our study presents the first direct evidence that inhibition of TAK1 mitigated MI/R injury, and TAK1 mediated ROS/ER stress/apoptosis signal pathway is important for the pathogenesis of MI/R injury.

**HIGHLIGHTS**

- TAK1 has an important role in myocardial ischaemia/reperfusion injury.
- Inhibition of TAK1 mitigates oxidative stress and ER stress to protect against myocardial ischaemia/reperfusion injury.
- The TAK1/ROS/ER stress pathway is important for the pathogenesis of myocardial ischaemia/reperfusion injury.

## 1 INTRODUCTION

Ischaemic heart disease is a common clinical cardiovascular disease that poses a serious threat to human health.^1^ Myocardial hypoxia is the basic pathological process of ischaemic cardiomyopathy. Long‐term hypoxia and malnutrition in the heart can result in cardiomyocyte death, leading to myocardial remodelling and heart failure.^1^, ^2^ In the clinical practice of ischaemic heart disease, myocardial ischaemia/reperfusion (MI/R) can improve blood supply to the ischaemic myocardium but can also lead to severe arrhythmia, myocardial dysfunction and myocardial stunning, and myocardial necrosis caused by cell necrosis or apoptosis can result in tissue necrosis.^3^, ^4^, ^5^, ^6^ In recent years, the incidence of MI/R injury has increased year by year.^2^ The underlying mechanisms of MI/R injury include free radical damage, calcium overload, energy metabolism disorder, leukocyte activation and microvascular damage, resulting in endoplasmic reticulum (ER) and mitochondrial function injury.^7^, ^8^, ^9^ However, the mechanism of MI/R injury has not been fully elucidated.

ER stress, oxidative stress and mitochondria dysfunction can lead to myocardial injury.^10^, ^11^, ^12^ During MI/R, glucose and nutrient deficiency, ATP depletion, large amounts of free radical reactive oxygen species (ROS) and destruction of Ca^2+^ homoeostasis lead to ER stress and ER dysfunction, causing unfolded proteins response (UPR), which further cause ER stress.^13^, ^14^, ^15^ Protein kinase RNA‐like endoplasmic reticulum kinase (PERK), inositol‐requiring enzyme 1 (IRE1) and activating transcription factor 6 (ATF6) are three sensor/mediator proteins in the ER.^16^ When stress response, these three proteins are separated from glucose‐regulated protein 78 (GPR78) and become active.^17^ It has been reported that prolonged and/or excessive ER stress induces ER‐related cell apoptosis^10^, ^18^, ^19^ including PERK‐dependent induction of C/EBP homologous protein (CHOP) and the IRE1α‐mediated activation of caspase 12 proteolytic enzyme activation.^20^, ^21^ Some studies have reported that MI/R‐induced cardiomyocyte dysfunction is consistent with changes in oxidative stress and endothelium‐dependent response.^22^ And ROS‐induced ER stress mediated cardiomyocyte apoptosis.^23^

Transforming growth factor β‐activated protein kinase 1 (TAK1) is a major member of the mitogen‐activated protein kinase (MAPK) family involved in various biological responses, including inflammation, apoptosis, differentiation and survival of different cell types.^23^, ^24^, ^25^ Once activated, TAK1 phosphorylates MAPK kinases MKK4 and MKK3/6, which activate p38 MAPK and JNK, respectively. In addition, TAK1 activates the NF‐κB pathway by interacting with TRAF6 and phosphorylating the NF‐κB inducing kinase.^26^ Tissue‐specific deletion of TAK1 results in severe cell death and tissue damage in liver, epidermis, endothelium and intestinal epithelial cells.^27^, ^28^, ^29^ Our previous study has also shown that TAK1 signalling pathway is involved in the regulation of cardiac hypertrophy.^23^, ^24^, ^25^ It has been reported that notoginsenoside R1 inhibits the activation of TGF‐β1‐TAK1 signalling pathway and protects the heart from rabbit lung remote ischaemia/reperfusion (I/R) injury.^30^ At the same time, Dusp14 prevents hepatic I/R injury by inhibiting TAK1.^31^ These results suggest that TAK1 plays an important role in the regulation of cardiomyocyte death and I/R injury. However, the role of TAK1 on MI/R injury in mice has not been fully determined.

In tumour cells, ablation of TAK1 in keratinocytes and Molt‐4 cells causes hypersensitivity to ROS‐induced cell apoptosis, while significantly increasing hyperthermia‐induced CHOP expression_._ ^32^, ^33^, ^34^ Kazuhito et al found that after treating Tak1‐deficient mouse fibroblasts and keratinocytes with ER stress inducers Tak1 deficiency increased cell survival, attenuated proteolytic cleavage of caspase 3 and Chop expression during ER stress.^17^ Furthermore, Daisuke et al reported that the inhibition of p38 MAPK activity resulted in a significant decrease in the production of ROS in cardiomyocytes.^35^ Therefore, we investigated the role of TAK1 in the regulation of MI/R and the effects of TAK1 on MI/R‐induced oxidative stress and ER stress.

## 2 MATERIALS AND METHODS

### 2.1 Methods

Male C57/B6 mice (8‐12 weeks of age) were purchased from the Animal Center of the Wenzhou Medical University. All mice were housed in a temperature‐controlled barrier facility with a 12‐hour light/dark cycle. The animal use and care procedures were in accordance with the Guide for the Care and Use of Laboratory Animals from the National Institutes of Health. Meanwhile, this study was approved by the Animal Care and Use Committee of Wenzhou Medical University (Number: wydw2014‐0058). MI/R injury model was constructed by myocardial ischaemia for 30 minutes, followed by reperfusion for 0, 1, 2, 3 or 4 hours. Mice were intubated and ventilated with isoflurane (1.5% v/w/) at 100‐120 respiratory rate and 200‐250 µL/min tidal volume. The distal 1/3 of the left anterior descending coronary artery (LAD) was ligated with 7‐0 silk suture, and reperfusion was performed for 4 hours after coronary artery occlusion for 30 minutes, and then, the ligation was released. Sham‐operated mice were anesthetized, and the suture was placed under the LAD of the mice, but without ligation.

### 2.2 Animal treatment

30 minutes before ischaemic surgery, the mice were intraperitoneally injected with the specific TAK1 inhibitor 5Z‐7‐oxozeaenol (5Z‐7‐ox; O9890‐1MG; Sigma, 5 mg/kg) as previously described by Wang X et al^31^ DMSO was injected intraperitoneally as the vehicle control. Furthermore, recombinant adeno‐associated virus serotype 9 (AAV9) vectors (Taitool Bioscience, Shanghai, China) harbouring TAK1 shRNA or Sh‐Con with eGFP and a c‐TNT promoter (AAV9‐cTNT‐eGFP‐Sh‐Con, AAV9‐cTNT‐eGFP‐Sh‐RNA) were employed to examine the effects of TAK1 silencing in MI/R injury. The sequence of DNA corresponding to TAK1 shRNA was AAGCAACAGAGTGAATCTGGA. AAV9‐cTNT‐eGFP‐Sh‐TAK1/ Sh‐Con vectors [3 × 10^11^ vector genomes (vg)/mouse] were injected through tail vein of mice. 5 weeks after AAV9 injection, TAK1 silencing was verified by PCR and Western blot.

### 2.3 Echocardiography

Transthoracic echocardiography was accomplished with an M‐mode transducer (15‐MHz linear transducer, Vevo1100, Visual Sonics, Toronto, Canada), as previously described.^25^ At the papillary muscle level, the left ventricular shortening (LVFS) and left ventricular ejection fraction (LVEF) were measured by recording the short‐axis view of M‐type traces on the left anterior and posterior walls.

### 2.4 Evans blue/TTC double‐staining

The myocardial infarct size was determined by Evan's blue and triphenyltetrazolium chloride (TTC) (Sigma‐Aldrich, St. Louis, MO, USA) double‐staining method. The LAD was ligated again after reperfusion, and 0.2 mL 2% Evan's blue dye was injected into the inferior vena cava. When the blue appears on the right side of the heart, the heart was quickly removed and rinsed with saline, frozen −20°C. Five 1mm thick heart sections were made and then were incubated in 1% TTC for 20 minutes at 37°C. After TTC staining, the red viable tissue was the area at risk (AAR), darker blue was the non‐ischaemic myocardium and the pale area was the infarct size (INF). The myocardial infarct size, AAR and left ventricular (LV) size of each slice were determined by Image‐Pro Plus software. The ratio of infarct size to AAR (expressed in%) and AAR to LV (expressed in%) was calculated.

### 2.5 Determination of cardiac troponin I (cTnI) levels

The cTnI levels in mice plasm were measured using commercial ELISA kits (Shanghai Westang Bio‐Tech Co., Ltd., Shanghai, China) following the instructions.

### 2.6 TUNEL staining

The heart tissues were then embedded in OCT (Optimum Cutting Temperature) compound and were cut into approximately 5 µm thick sections. Then, the sections were subjected to the TUNEL (Roche, Mannheim, Germany) staining to detect cardiomyocyte apoptosis according to the kit's manual. The section images were taken by a Nikon ECLIPSE Ti microscope (Olympus BX51, Tokyo, Japan).

### 2.7 Oxidative stress detection in the myocardium

DHE staining was used to detect the accumulation of ROS in myocardium. For in situ DHE staining, fresh frozen cardiac cross section (5 μm thick) was covered with DHE (5 μM) in a 37°C incubator for 30 minutes and then washed with phosphate‐buffered saline (PBS) for 5 minutes to remove unbounded dyes. The malondialdehyde (MDA) levels and superoxide dismutase (SOD) activity were measured in mice heart using commercial reagent kit (JianCheng Bioengineering Institute, Nanjing, China) according to the instructions.

### 2.8 Hypoxia/reoxygenation (H/R) model

Murine neonatal cardiomyocytes were isolated from 1‐3‐day‐old neonatal C57/B6 mice hearts as previously described by Ehler E. and colleagues.^36^ Subsequently, cardiomyocytes (CMs) were used to induce hypoxia reoxygenation injury model in vitro. The cells were cultured with glucose‐free DMEM (GIBCO, 11966‐25, Carlsbad, CA, USA) in a tri‐gas incubator with 95% N2 and 5% CO2 for 6 hours. Then, the cells were grown under normal culture conditions for 2, 4 or 6 hours to induce the reoxygenation injury.^22^

### 2.9 Cell transfection

To inhibit TAK1 expression, CMs were transfected with small interfering RNA (siRNA) against TAK1 conjugated (Guangzhou RiboBio, Guangzhou, China) with Lipofectamine 3000 (Invitrogen, Carlsbad, CA, USA) for 6 hours. Cells were harvested 48 hours after transfection and used for further analysis. Adenovirus vectors for TAK1 overexpression and control GFP vector were commercially purchased from GeneChem Co., Ltd (Shanghai, China) and transfected with 20 MOI. The cells were treated with 10 mmol/L ROS scavenger N‐acetylcysteine (NAC; A9165‐5G; Sigma, St. Louis, MO, USA) during H/R.

### 2.10 Cell viability

The number of viable CMs was evaluated by MTS assay (Promega, Beijing, China). Briefly, 20% MTS dye solution was added to each well and incubated for 2.5 hours. The number of viable cells was determined by evaluating absorbance at 480 nm. The MTS assay was repeated three times.

### 2.11 Determination of apoptosis ratio

The CMs were collected and incubated with FITC annexin V Apoptosis detection kit (cat#556547, BD Biosciences, Franklin Lakes, NJ, USA) or PE annexin V Apoptosis detection kit (cat#559763, BD Biosciences, USA).The flow cytometry was used to detect the apoptosis, and BD FACS software was to quantify the apoptosis ratio; the experiments were repeated three times.

### 2.12 ROS detection assay

After different treatments, the CMs were exposed to 50 μM DCFDA (Sigma‐Aldrich, St. Louis, MO, USA) for 30 minutes at 37°C in darkness, and then cells were collected and washed twice with PBS. The green fluorescence of cells was analysed by a flow cytometry within 1 hour. The above experiments were repeated three times.

### 2.13 Immunofluorescence staining

The heart tissues were embedded in OCT compound and cut approximately 5 µm thick sections. For immunofluorescence, slides were permeated with 0.5% Triton X‐100 for 10 minutes and blocked with goat serum at room temperature for 1 hour. Then, they were incubated with an antibody targeting anti‐p‐TAK1 (1:100, Cell Signaling Technology). The slides were washed and followed by a further incubation at room temperature for 1 hour with an AlexaFluor®488 Goat anti‐Rabbit IgG (H+L) (cat# ab150077, Abcam) at 2 µg/mL and Phalloidin‐iFluor 594 reagent (1:100, cat# ab176757, Abcam). Nuclear DNA was labelled in blue with DAPI. Cells were fixed with 100% methanol (5 minutes) and then blocked in 1% BSA in 0.1%PBS‐Tween for 1 hour. The cells were then incubated with CHOP (L63F7) (1:100, cat# 2895, Cell Signaling Technology) overnight at +4°C, followed by a further incubation at room temperature for 1 hour with an AlexaFluor®488 Goat anti‐Mouse secondary (cat# ab150117, Abcam) at 2 µg/mL. Nuclear DNA was labelled in blue with DAPI. The results were calculated blindly by counting the positive staining cells in 10 high‐power field of vision (HPF)/sections.

### 2.14 Real‐time quantitative PCR

Total RNA was isolated from the cells using TRIzol reagent (Invitrogen, Carlsbad, CA, USA) according to the manufacturer's instructions. The reverse transcription PCR was performed by using TransScript One‐Step gDNA Removal and cDNA Synthesis SuperMix (Transgen BioTech, Beijing, China) according to the manufacturer's instruction. RT‐PCR was performed using IQ SYBR Green Supermix (Bio‐Rad, Hercules, CA, USA), and mRNA expression was normalized to β‐ACTIN expression. The following primers were used for analysis:

mou β‐ACTIN forward primer:5′‐TGAGCTGCGTTTTACACCCT‐3′

mou β‐ACTIN reverse primer:5‐TTTGGGGGATGTTTGCTCCA‐3′

mouTAK1 forward primer:5‐CAGATGGAATTACAGGACTATG‐3′

mou TAK1 reverse primer:5‐GGTAGGCGGACAAGAATC‐3′

### 2.15 Western blot

The total protein was extracted and purified, and then the concentration was determined by a BCA Protein Assay Kit (Beyotime, Shanghai, China). 50 µg protein was separated by 10%‐20% gel and transferred onto a PVDF membrane (Thermo Fisher, Waltham, MA, USA). Then, the membrane was incubated with relevant primary antibodies overnight, followed by the incubation with the corresponding secondary antibodies (mouse or rabbit antibody, 1:5000, Cell Signaling Technology). The amount of the proteins was analysed using Image‐Pro Plus and normalized to their respective controls. The antibodies used were as follows: anti‐TAK1 (1:1000,cat# ab109526, Abcam), anti‐GRP78 (C50B12) (1:1000, cat# 3183, Cell Signaling Technology), anti‐ATF6 (1:1000; cat# ab37149, Abcam), anti‐PERK (C33E10) (1:1000, cat# 3192, Cell Signaling Technology), anti‐p‐PERK (Thr980) (1:1000, cat# MA5‐15033, Invitrogen), anti‐IRE1α (14C10) (1:1000, cat# 3294, Cell Signaling Technology), anti‐p‐IRE1α (phospho S724) (1:1000, cat# ab48187, Abcam), anti‐p‐eIF2α (Ser51)(D9G8) (1:1000, cat# 3398, Cell Signaling Technology), anti‐eIF2α (D7D3) (1:1000, cat# 5324, Cell Signaling Technology), anti‐ATF4 (1:1000; cat# ab23760, Abcam), anti‐Caspase12 (1:1000, cat# 2202, Cell Signaling Technology), anti‐Caspase 3 (1:1000, cat# 9662, Cell Signaling Technology), anti‐Cleaved Caspase 3 (Asp175) (1:1000, cat# 9664, Cell Signaling Technology), anti‐JNK (1:1000, cat# 9252, Cell Signaling Technology), anti‐p‐JNK (Thr183/Tyr185) (81E11) (1:1000, cat# 4668, Cell Signaling Technology), anti‐β‐actin (13E5) (1:1000, cat# 4970, Cell Signaling Technology), anti‐GAPDH (14C10) (1:1000, cat# 2118, Cell Signaling Technology).

### 2.16 Statistical analysis

SPSS 22 software (Unicom, Mosson Hills, CA, USA) was used for statistical analyses. All data were expressed as the mean ± SEM. For data with variance homogeneity, all outcomes among groups were compared using a one‐way ANOVA, followed by the Dunnett multiple comparison test. A value of *P* < 0.05 was considered significant.

## 3 RESULTS

### 3.1 TAK1 activation is associated with MI/R injury

**FIGURE 1:**
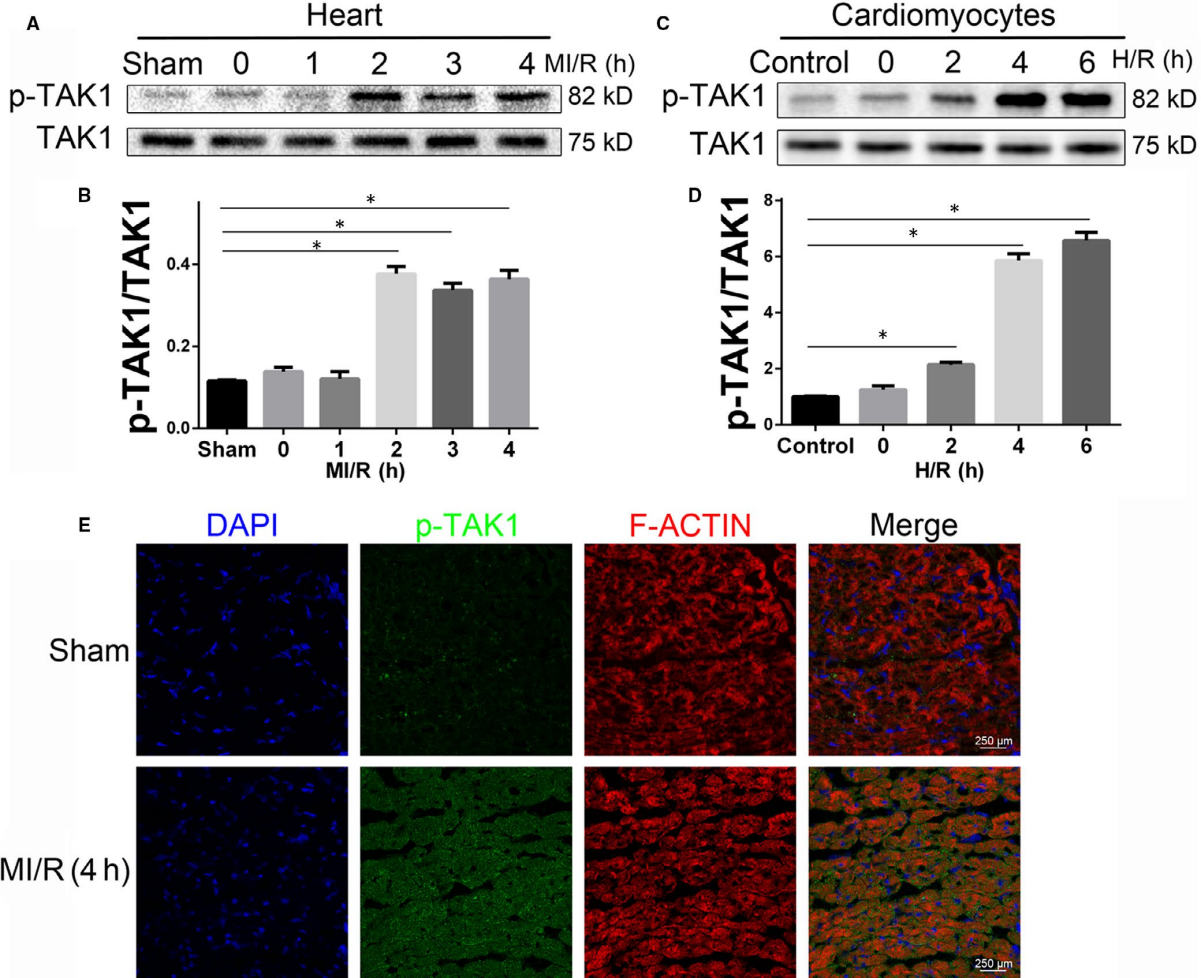
P‐TAK1 up‐regulation coincides with MI/R injury. A Western blot analysing the p‐TAK1 protein level in hearts from mice subjected to sham treatment or ischaemic for 0.5 hour followed by reperfused for oh, 1, 2, 4 hours (n = 6 per time point). *Indicates *P* < 0.05 compared with the sham group; (B) the quantitative analysis of (A); (C) the expression level of p‐TAK1 expression in cardiomyocytes after sham or H/R treated (n = 6 per time point). *Indicates *P* < 0.05 compared with the control group; (D) the quantitative analysis of (C); (E) representative immunofluorescent image of p‐TAK1 (green) and F‐ACTIN (red) in the border zone of reperfused myocardium 4 hours after reperfusion (n = 6/group), all nuclei were stained with DAPI (blue), magnification, ×400. Data are shown as means ± SEM

To investigate the potential role of TAK1 in MI/R injury, we measured TAK1 expression in the cardiac pathological model. The expression of p‐TAK1 was gradually up‐regulated from 1 to 4 hours post MI/R (Figure 1A,B). Subsequent experiments were performed at 4 hours of reperfusion. And the mRNA level of TAK1 was not altered after MI/R injury (Figure S1). And a gradual up‐regulation of p‐TAK1 expression was detected after reoxygenation at 2, 4 and 6 hours in CMs subjected to H/R insult (Figure 1C,D). Subsequent experiments were conducted at 6 hours of reoxygenation. Immunofluorescence staining revealed a significant increase in p‐TAK1 in the ischaemic and reperfused area (Figure 1E). These results indicated that MI/R injury promoted the activation of TAK1, suggesting that TAK1 might play an important role in MI/R injury.

### 3.2 Inhibition of TAK1 ameliorates myocardial dysfunction and myocardial injury

**FIGURE 2:**
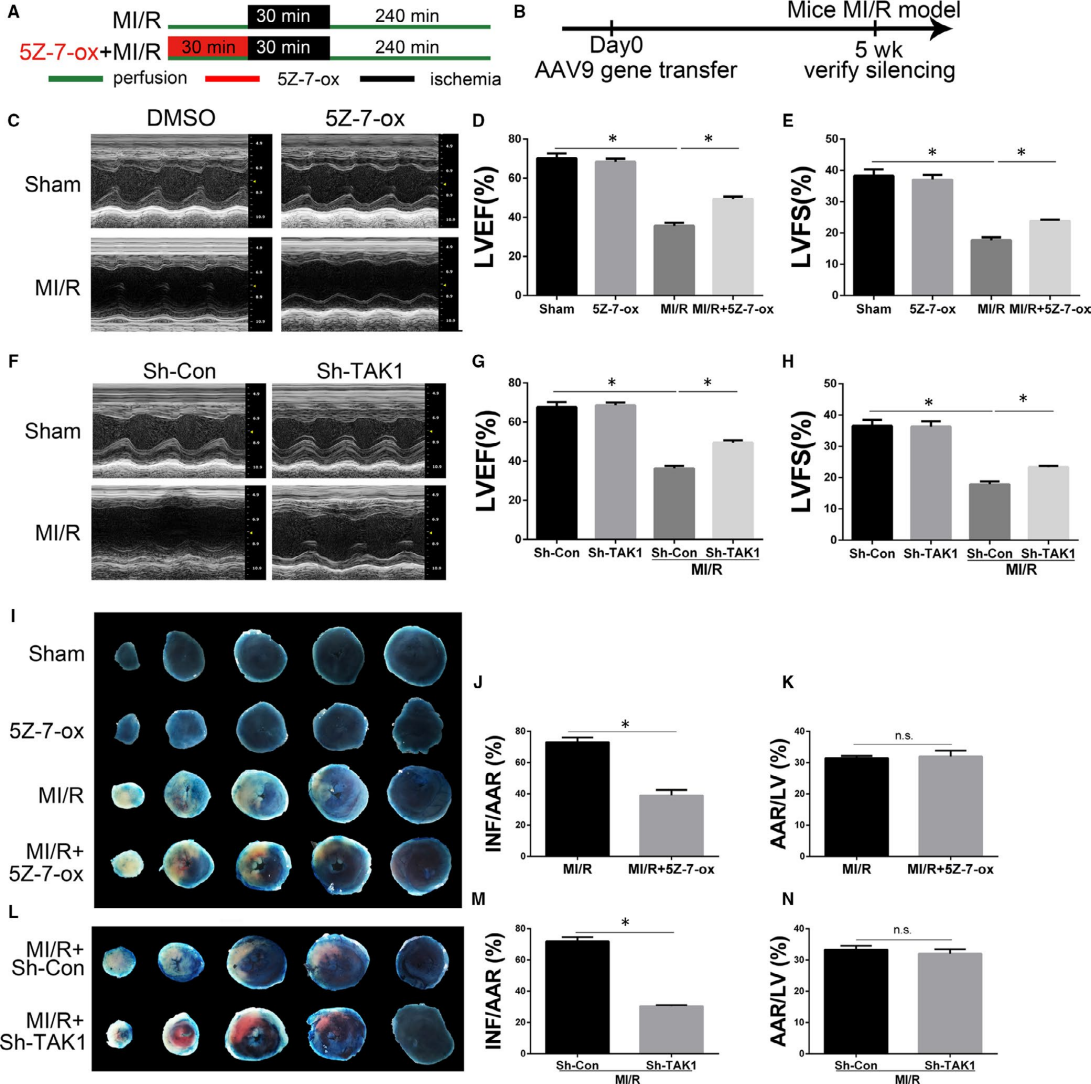
The effects of TAK1 silencing on MI/R‐induced myocardial dysfunction and myocardial injury. A, Schematic illustration of the 5Z‐7‐oxozeaenol (5Z‐7‐ox) pretreatment protocol; (B) the time course of AAV9‐mediated silence of TAK1; (C) representative M‐mode echocardiogram image after 4 hours reperfusion in mice allocated to sham, 5Z‐7‐ox, MI/R, MI/R+5Z‐7‐ox groups; (D) the quantitative analysis of left ventricular ejection fraction (LVEF) based on (C) (n = 6/group); (E) the quantitative analysis of left ventricular fractional shortening (LVFS) based on (C) (n = 6‐8/group); (F) representative M‐mode echocardiogram image (n = 6/group); (G) the quantitative analysis of LVEF (n = 6/group) and (H) LVFS (n = 6/group) based on (F); (I) representative photographs of Evans blue/TTC‐stained sections in mice allocated to sham, 5Z‐7‐ox, MI/R, MI/R+5Z‐7‐ox groups (n = 6/group), infarct area (INF: white), area at risk (AAR: red and white), and normal area (blue); (J) ratio of infarct size to area at risk (n = 6/group) based on (I); (K) ratio of area at risk to left ventricular (n = 6/group) based on (I); (L) representative Evans blue/TTC double‐stained LV transverse slices after 4 hours reperfusion in mice allocated to Sh‐Con, Sh‐TAK1‐treatment groups (n = 6/group); (M) ratio of infarct size to area at risk (n = 6/group) and (N) area at risk to left ventricular (n = 6/group) based on (L). Data are shown as means ± SEM; *P < 0.05, between the indicated groups

**FIGURE 3:**
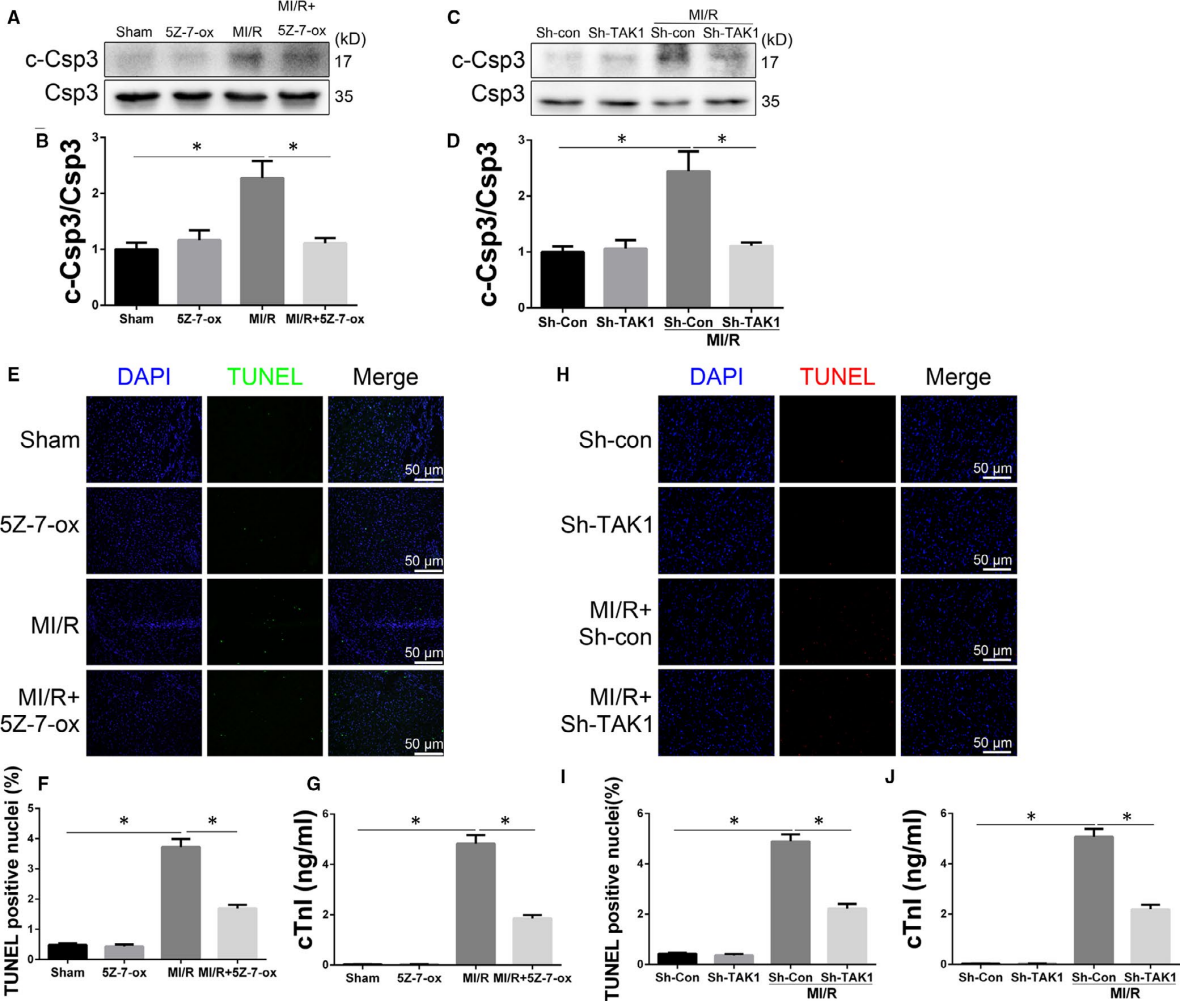
TAK1 silencing alleviated MI/R injury. A, Western blot analysing the cardiac caspase 3 activity level in the border zone 4 hours post perfusion from mice subjected (n = 6/group); (B) the quantitative analysis of (A); (C) Western blot analysing the cardiac caspase 3 activity level (n = 6/group); (D) the quantitative analysis of (C); (E) representative cardiac TUNEL staining (green) to reveal cardiac apoptosis in the border zone 4 hours post perfusion(n = 6/group), magnification, ×400; (F) the apoptosis index (TUNEL positivity) in cardiac section, a quantification of (E); (G) cardiac troponin I (cTnI) level in mice serum 4 hours post perfusion (n = 6/group); (H) representative cardiac TUNEL staining (red) to reveal cardiac apoptosis (n = 6/group), magnification, ×400; (I) the apoptosis index in cardiac section, a quantification of (H); (J) cTnI leaves in mice serum (n = 6/group). Data are shown as means ± SEM; *P < 0.05, between the indicated groups

In order to investigate the role of TAK1 in MI/R, the specific TAK1 inhibitor 5Z‐7‐ox was intraperitoneally injected into mice 30 minutes before ischaemia (Figure 2A).^31^ To further clarify the role of TAK1 in the MI/R injury, we employed AAV9 vector with a cTnT promoter to selectively silence TAK1 in cardiomyocytes. As shown in Figure 3B, five weeks after AAV9 vector delivery, mice were then subjected to MI/R operation. The inhibitory effect of Sh‐TAK1 on cardiac TAK1 was confirmed by PCR and Western blot (Figure S2). To determine cardiac function, non‐invasive echocardiography was employed. MI/R induced remarkable left ventricular dysfunction, as evidenced by decreased LVEF and LVFS, whereas 5Z‐7‐ox treatment significantly improved LVEF and LVFS (Figure 2C‐E). Furthermore, compared with MI/R+Sh‐Con group, selectively silence TAK1 in cardiomyocytes also improved of LVEF and LVFS (Figure 2C‐E). Moreover, the AAV9‐cTNT‐eGFP vector carrying shTAK1 displayed no additional effect on cardiac performance in the basic state. Myocardial infarct size was measured by TTC staining. As shown in Figure 2I, the red area represented ischaemic myocardial tissue, while the white represented infarction region. Obviously, no infarction was found in myocardial tissue slices in the sham group and 5Z‐7‐ox group. The myocardial infarction area pretreated with 5Z‐7‐ox was obviously smaller than that of the MI/R group (Figure 2J), but the ischaemic area was similar (Figure 2K). Furthermore, compared with MI/R+Sh‐Con group, administration of Sh‐TAK1 significantly reduced infarct size, but the ischaemic area was similar (Figure 2L‐N). These findings corroborated that TAK1 silencing ameliorated myocardial dysfunction and alleviated myocardial injury.

### 3.3 TAK1 silencing alleviates cell damage induced by MI/R

Cardiomyocyte loss caused by MI/R injury has been considered to be due to necrotic cell death. But in the past decades, studies have found that myocardial cell loss is mainly attributed to apoptosis after myocardial infarction.^37^ To understand why TAK1 inhibition can reduce MI/R injury, Western blot was performed to analysis the cardiac caspase 3 activity level in the border zone from mice allocated to sham, 5Z‐7‐ox, MI/R, MI/R+5Z‐7‐ox groups (Figure 3A). Cleaved caspase 3 expression was up‐regulated by MI/R. However, 5Z‐7‐ox treatment reduced cleaved caspase 3 expression (Figure 3B). Moreover, compared with MI/R+Sh‐Con group, TAK1 silencing significantly suppressed caspase 3 activity (Figure 3C,D). TUNEL staining was performed in the perimyocardial infarction area (Figure 2E). A large number of TUNEL‐positive cardiomyocytes were observed in the MI/R group, while pretreatment with 5Z‐7‐ox reduced the number of TUNEL‐positive cardiomyocytes ((Figure 2F). We then examined cardiac troponin I (cTnI) in mouse plasm and found that the level of cTnI was increased after MI/R (Figure 2G), whereas 5Z‐7‐ox pretreatment significantly reduced the MI/R‐induced release of cTnI in the plasm. Furthermore, compared with MI/R+Sh‐Con group, TAK1 silencing reduced TUNEL‐positive cardiomyocytes (Figure 2H,I) and decreased serum level of cTnI (Figure 3J). These findings confirmed that TAK1 silencing alleviated cardiac apoptosis and MI/R injury.

### 3.4 TAK1 silencing mitigates oxidative stress and dissipates ER stress

**FIGURE 4:**
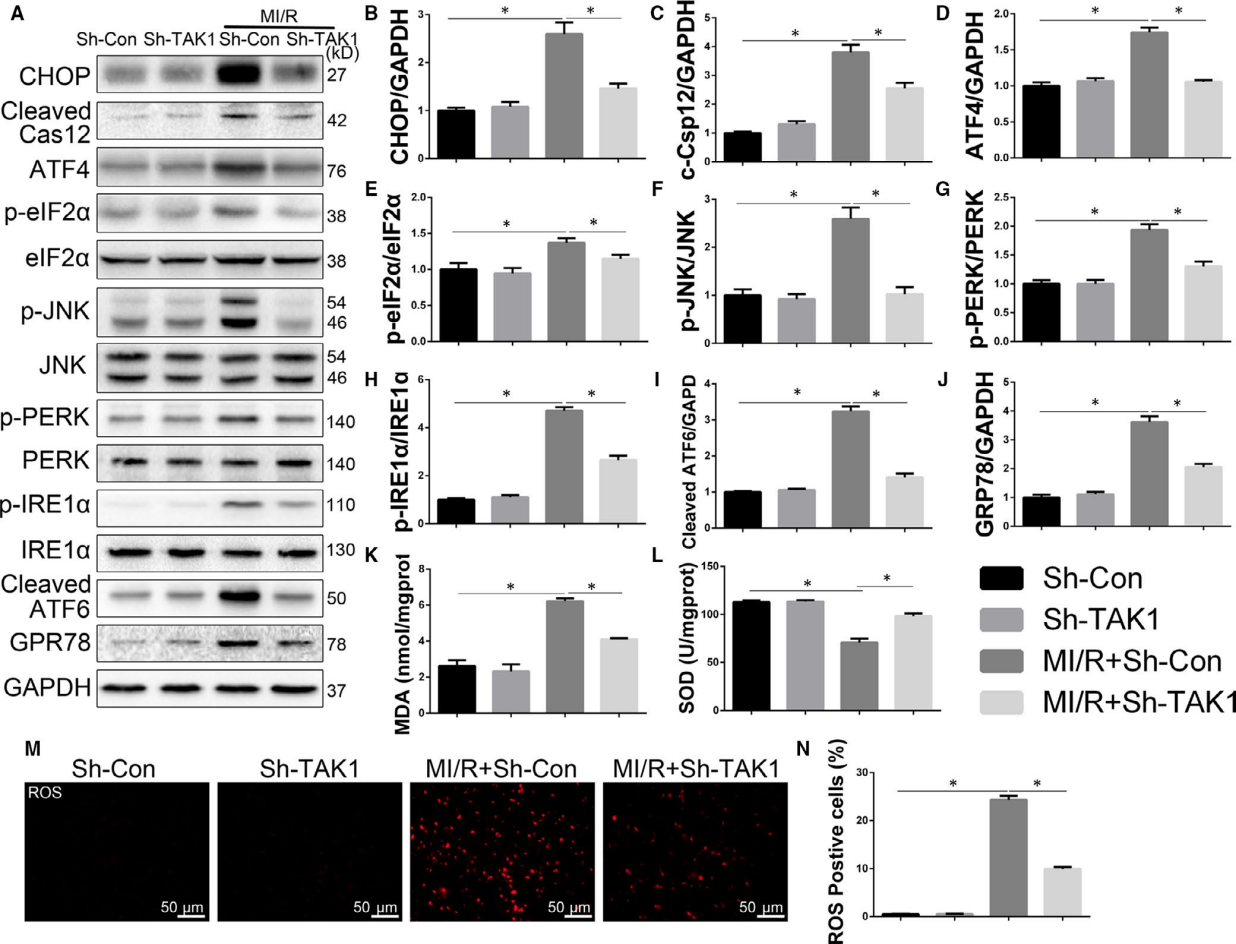
TAK1 silencing mitigated oxidative stress and dissipated ER stress in MI/R‐injured myocardium. A through L, The protein expression levels and optical density analysis of CHOP, cleaved caspase 12, ATF4, p‐eIF2α, p‐JNK, p‐PERK, p‐IRE1α, cleaved ATF‐6 and GRP‐78 in the myocardium (n = 6/group); (K) the concentration of malondialdehyde (MDA) and (L) the activity of superoxide dismutase (SOD) in myocardium (n = 6/group); (M) DHE staining and evaluate (N) the amount of ROS in cardiac tissue (n = 6/group), magnification, ×400; Data are shown as means ± SEM; *P < 0.05, between the indicated groups

MI/R injury affects the ER integrity and promotes ER stress.^16^ To further determine the mechanisms of TAK1 silencing reducing cardiac apoptosis, the influences of TAK1 silencing on CHOP (index of ER stress apoptosis) and caspase 12 (index of ER stress‐required apoptosis) were examined. As shown in Figure 4A‐C, CHOP and cleaved caspase 12 expression were significantly up‐regulated after MI/R when compared to Sh‐Con group (*P* < 0.05). However, TAK1 silencing attenuated CHOP and cleaved caspase 12 expression (*P* < 0.05). Importantly, TAK1 silencing did not change the expression of such proteins under baseline condition. Three main sensor proteins in the ER were also examined. TAK1 silencing attenuated MI/R‐induced ER stress markers including PERK, IRE1α and cleaved‐ATF6 (Figure 4G‐I). Western blots were also employed to examine some ER stress markers. Western blots confirmed that TAK1 silencing attenuated MI/R‐induced ER stress markers including GRP78, p‐eIF2α, ATF4 and p‐JNK (Figure 4D‐J).

And oxidative stress has been proven to be involved in metabolic syndrome and numerous cardiovascular diseases. To decipher further the molecular mechanisms lying behind the ER‐mediated protective actions of TAK1 silencing, we investigated the effects of TAK1 silencing on oxidative stress. The level of MDA (Figure 4K, index of lipid oxidative product) was markedly increased after MI/R, while TAK1 silencing significantly reduced the content of MDA in cardiac tissue. On the contrary, the level of SOD was decreased after MI/R. Moreover, the content of SOD in TAK1 silencing group was higher than that in the MI/R+Sh‐Con group and had a tendency to return to the normal (Figure 4L). DHE staining was performed to evaluate the content of ROS in cardiac tissue. As shown in Figure 4M,N, ROS production increased after MI/R, whereas TAK1 silencing disrupted MI/R‐induced ROS production. Taken together, these results suggested that TAK1 silencing attenuated oxidative stress in the reperfused myocardium and subsequently mitigated the apoptotic pathways mediated by ER stress.

### 3.5 Down‐regulation of TAK1 activation attenuates H/R injury in neonatal cardiomyocytes

**FIGURE 5:**
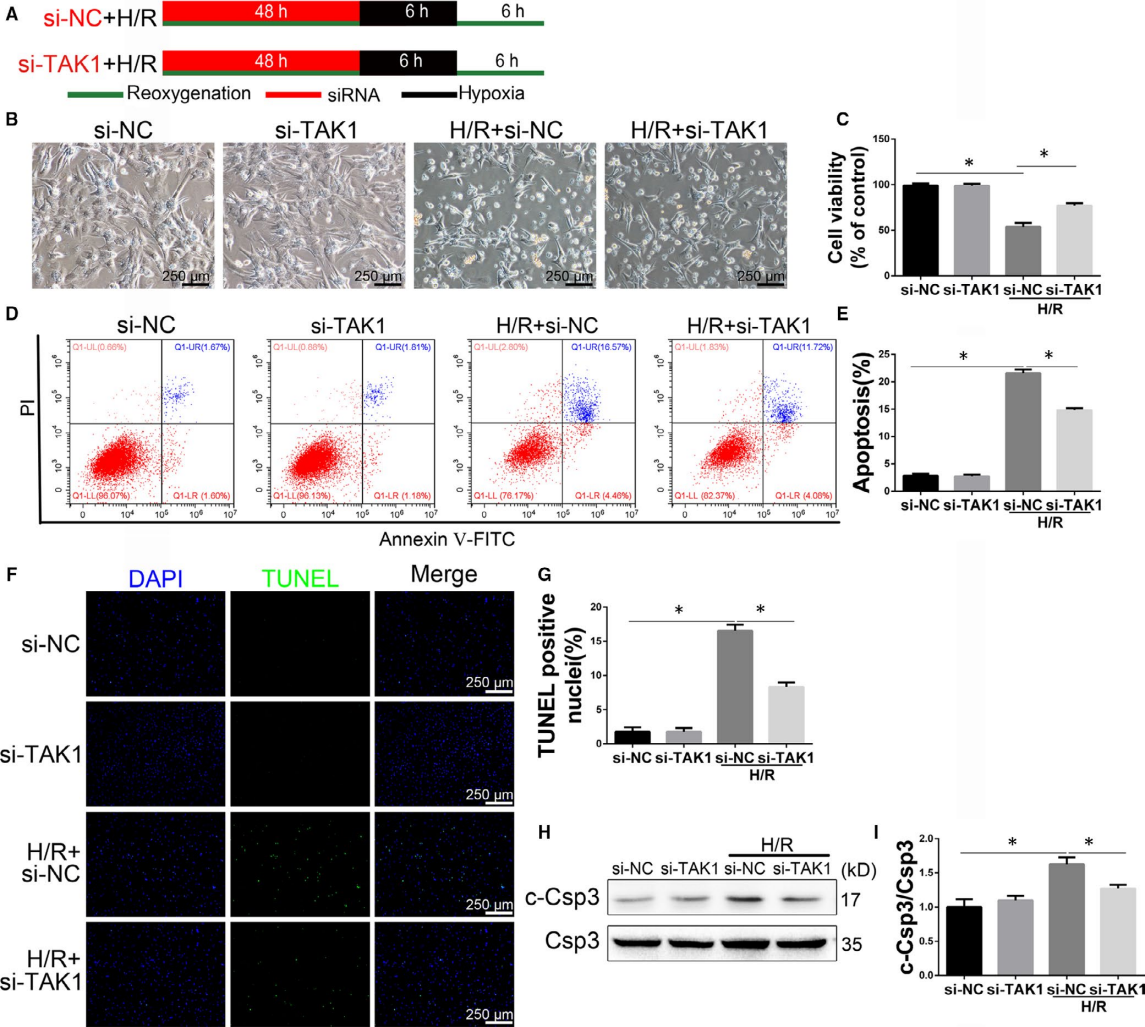
Transfecting cells with si‐TAK1 attenuated hypoxia/reoxygenation (H/R) injury in neonatal cardiomyocytes. A, Schematic illustration of the protocol used for H/R injury in neonatal cardiomyocytes (CMs); (B) representative morphology image after H/R in CMs allocated to si‐NC, si‐TAK1, H/R+si‐NC, H/R+si‐TAK1 groups, magnification, ×100; (C) cell viability was assessed by the MTS assay (n = 6/group); (D) CMs were collected and stained with annexin V‐FITC and PI for flow cytometry (n = 6/group); (E) quantification of total apoptotic (annexin V+) cells after FACS analysis (n = 6/group); (F) representative images of TUNEL in CMs after H/R injury (n = 6/group), magnification, ×100; (G) the quantitative analysis of (F). G, Western blot analysing caspase 3 activity level in CMs (n = 6/group); (H) the quantitative analysis of (G). Data are shown as means ± SEM; *P < 0.05, between the indicated groups

In order to further explore roles of TAK1 in MI/R injury, we employed hypoxia/reoxygenation (H/R)‐injured neonatal cardiomyocytes. We evaluated TAK1 function by transfecting cells with si‐TAK1 (Figure 5A). Inhibitory effect of si‐TAK1 was confirmed by PCR and Western blot (Figure S3). The CMs morphology was shown in Figure 5B. As shown in Figure 5C, silencing TAK1 attenuated H/R injury as evidenced by increased cell viability. PI and annexin V‐FITC staining were performed on cells, and then, FACS was used to quantify total apoptotic cells (annexin V+). The H/R‐induced increases in the proportions of total apoptotic cells were significantly inhibited by the transfection with si‐TAK1 (Figure 5D,E). Moreover, immunofluorescence demonstrated that H/R group exhibited more TUNEL‐positive cells than control group, but H/R+si‐TAK1 group obviously had fewer TUNEL‐positive cells than H/R+si‐NC group (Figure 5F,G). Western blot was performed to analyse the caspase 3 activity level in CMs (Figure 5H). Cleaved caspase 3 expression was up‐regulated by H/R. Compared with H/R+si‐NC group, TAK1 silencing significantly suppressed caspase 3 activity (Figure 3I). Our results suggested that decreased TAK1 activation attenuated H/R‐induced injury and may play a protective role in apoptosis in CMs.

### 3.6 Decreased TAK1 activation attenuates ROS production and ER stress‐mediated apoptosis induced by H/R

**FIGURE 6:**
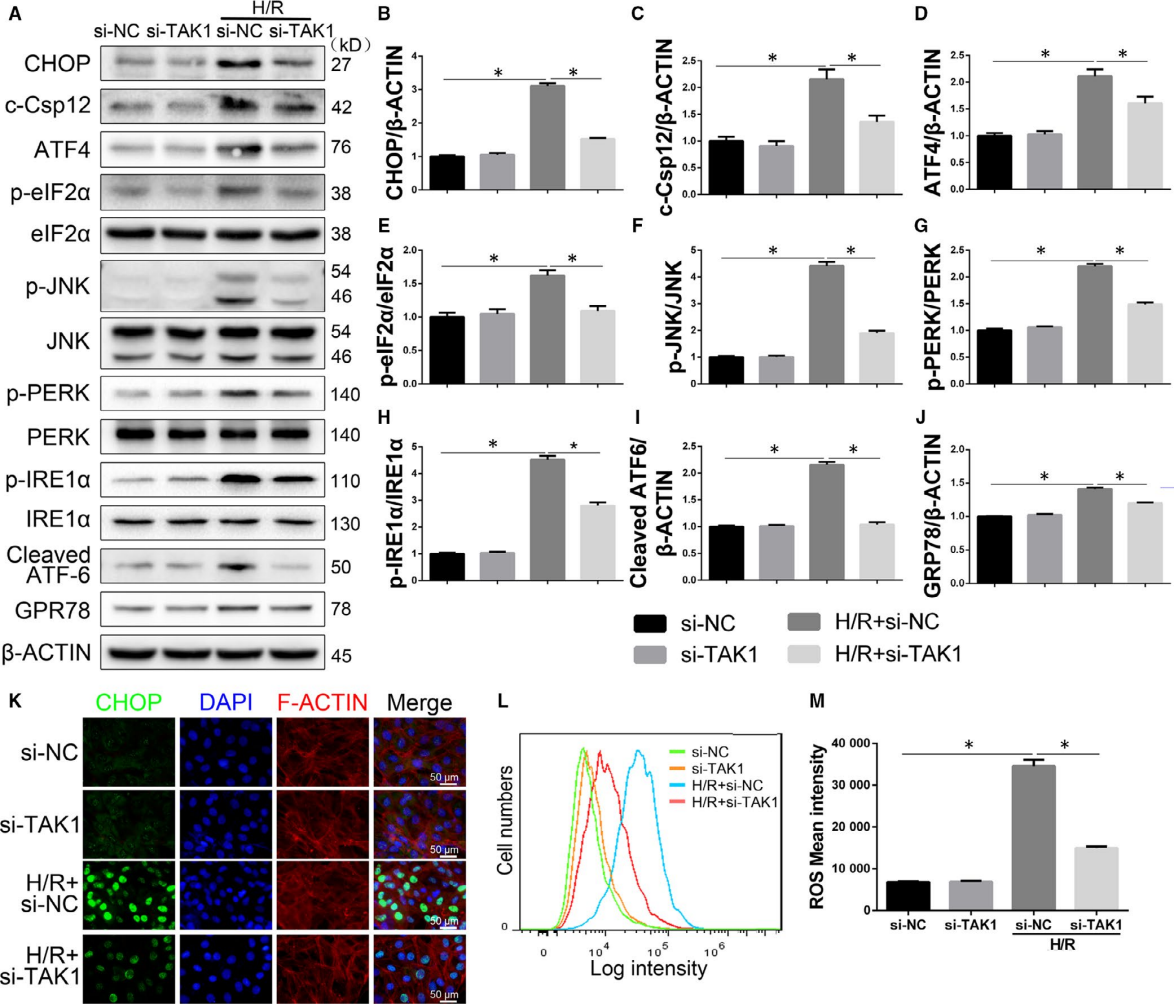
Decreased TAK1 activation attenuates ROS generation and ER stress‐mediated apoptosis in H/R‐injured cardiomyocytes (CMs). A through J, The protein expression levels and optical density analysis of CHOP, cleaved caspase 12, ATF4, p‐eIF2α, p‐JNK, p‐PERK, p‐IRE1α, cleaved ATF‐6 and GRP‐78 in CMs (n = 6/group); (K) representative immunofluorescent image of CHOP (green) and F‐ACTIN (red) in CMs, all nuclei were stained with DAPI (blue) (n = 6/group), magnification, ×400; (L) ROS generation was evaluated with flow cytometry using DCF fluorescence (n = 6/group); (M) the fluorescence intensity was calculated. Data are shown as means ± SEM; *P < 0.05, between the indicated groups

To elucidate the detailed mechanism of TAK1 on cardiomyocyte apoptosis and MI/R, we examined the level of ER stress‐related protein activity. As shown in Figure 6A‐J, compared with the si‐NC group, the expression of CHOP, cleaved caspase 12, ATF4, p‐eIF2α, p‐JNK, p‐PERK, p‐IRE1α, cleaved ATF‐6 and GRP‐78 was higher in H/R group. TAK1 silencing significantly inhibited the activation of ER stress‐related proteins induced by H/R. Importantly, the expression of such proteins in si‐TAK1 group was similar to si‐NC group. Immunofluorescence showed that H/R induced CHOP translocation from the cytoplasm to the nucleus (Figure 6K). The H/R group exhibited more CHOP‐positive cells than the si‐NC group, but the H/R+si‐TAK1 group had obviously fewer CHOP‐positive cells than the H/R group. As shown in Figure 6L,M, the fluorescence intensity of ROS in the H/R group was significantly higher than that in the si‐NC group, while that in the H/R+si‐TAK1 group was significantly decreased. These data suggested that ROS and the ER stress pathway were involved in the damage effect of TAK1 in CMs.

### 3.7 Inhibition of ROS partially reverses the damage effect of TAK1

**FIGURE 7:**
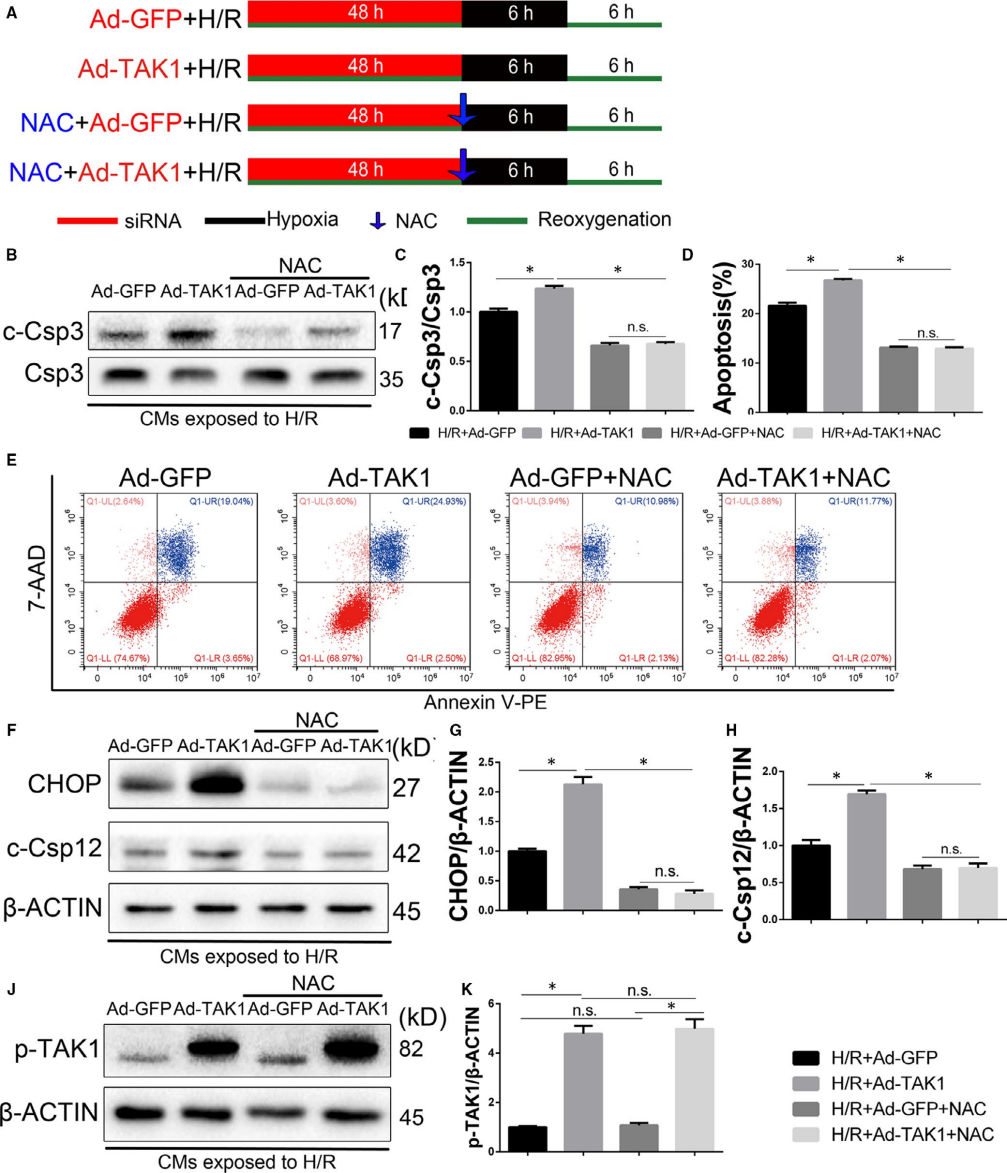
Inhibition of ROS attenuates TAK1‐mediated increase of myocardial apoptosis in the neonatal cardiomyocytes (CMs). A, Schematic illustration of the protocol used for hypoxia/reoxygenation Injury in CMs; (B) Western blot analysing caspase 3 activity in CMs (n = 6/group); (C) the quantitative analysis of (B); (D) and (E) annexin V‐PE and 7‐AAD stained CMs were detected by flow cytometry, followed by quantitative analysis of the total apoptotic cells (annexin V+) (n = 6/group); (F through H) The protein expression levels and optical density analysis of CHOP and cleaved caspase 12 in the CMs (n = 6/group); (I) Western blot analysis of p‐TAK1 expression in CMs (n = 6/group); (J) the quantitative analysis of (I). Data are shown as means ± SEM; *P < 0.05, between the indicated groups

To further confirm the relationship between the TAK1 and ROS generation, TAK1‐overexpressed neonatal CMs were exposed to H/R injury and pretreated with ROS inhibitor NAC (Figure 7A). As shown in Figure 7B‐E, compared with the H/R+si‐NC group, TAK1 overexpression increased caspase 3 activity and total apoptotic CMs that had been induced by H/R. Notably, ROS inhibition significantly protected CMs from the injury and normalized the H/R injury aggravated by TAK1 overexpression, which was manifested as suppressed caspase 3 activity and decreased total apoptotic CMs.

Signal transduction pathways involving CHOP and caspase 12 are known to mediate ER stress‐associated apoptosis. As shown in Figure 7F‐H, compared with the H/R group, the protein expression of CHOP and cleaved caspase 12 was increased in the TAK1 overexpression H/R group, whereas treated with NAC reduced the increased expression levels of these proteins. In addition, NAC treatment did not change the expression of p‐TAK1 under baseline and H/R condition (Figure 7I,J). These observations suggested that ROS inhibition eliminates the damage effect of TAK1 in H/R injury. Thus, our data suggest that ROS‐mediated ER stress mediates TAK1 function in myocardial during H/R injury.

## 4 DISCUSSION

Coronary heart disease is a global health problem involving blood flow recovery and acute myocardial infarction.^1^, ^2^ Studies have shown that oxidative stress and sudden metabolic changes can lead to myocardial injury.^37^ Our current study gains novel insights into the role of TAK1 in the heart. (a) The activation of TAK1 was significantly increased after MI/R, (b) Cardiomyocytes specific TAK1 silencing via AAV9 transfection ameliorated MI/R injury, (c) Inhibition of TAK1 mitigated oxidative stress and dissipated ER stress‐mediated apoptosis, thus reducing MI/R injury, (d) The inhibition of the ROS by specific inhibitor NAC partially reversed the damage effect of TAK1. These results indicate that TAK1 increases MI/R‐induced myocardium damage by activating ER stress‐mediated apoptosis and its function depends on ROS production.

TAK1 is an important signalling protein that activates a variety of signalling pathways in response to growth factors, cytokines and microbial products.^38^, ^39^, ^40^ Activation of TAK1 is tightly regulated through its binding partners and protein modifications. In vivo activation of TAK1 requires association with TAK1 binding protein 1 (TAB1), which triggers phosphorylation of TAK1.^41^, ^42^ Another adaptor protein, TAB2, links TAK1 with TRAF6 and mediates TAK1 activation upon IL‐1 stimulation.^43^ K63‐linked polyubiquitination at the K158 residue of TAK1 by TRAF6/ UBC13/UEV1A is an important response to stimulation of cells by cytokines and microbial products.^44^, ^45^ TAK1 polyubiquitination induces autophosphorylation at Thr187,^46^ its activation loop and other sites, including Thr184 and Ser192.^47^ It has been reported that TAK1 inhibition attenuates early brain injury and improves neurological deficits after Experimental Subarachnoid Hemorrhage.^48^ And Dusp14 prevents hepatic I/R injury by inhibiting TAK1.^31^ However, whether TAK1 is associated with the heart protection in the MI/R models remains largely unknown. The present study showed that the activation of TAK1 was significantly increased after MI/R, and the inhibition of TAK1 activation could reduce H/R injury in neonatal CMs. In vivo, TAK1 silencing by 5Z‐7‐ox or AAV9‐cTNT‐eGFP‐sh‐TAK1 could alleviate infarct size and cardiac tissue cells apoptosis. Thus, inhibition of TAK1 may protect MI/R injury via maintaining the survival of myocardial cells.

Perturbation of ER‐associated functions results in ER stress via UPR, leading to up‐regulated expression of ER resident chaperones, inhibition of protein synthesis and activation of protein degradation.^15^, ^16^ GRP78 binds to several critical transmembrane ER signalling proteins, including ATF6, PERK and IRE1. During ER stress, these transmembrane proteins are released from GRP78 and initiate specific apoptosis pathway including the transcriptional induction of CHOP as well as expression of cleaved caspase 12.^15^, ^16^ Kazuhito et al found that Tak1 deficiency increased cell survival, attenuated proteolytic cleavage of caspase 3 and Chop expression during ER stress in mouse fibroblasts and keratinocytes.^17^ Furthermore, panaxydol induces apoptosis in cancer cells through activation of TAK1 and p38/JNK, and p38 and JNK activate NADPH oxidase, the resulting oxidative stress triggered ER stress.^14^ Therefore, we explored the role of TAK1 in the mechanism of MI/R‐induced ER stress. This study showed that decreased TAK1 activation led to the reduced expression of GRP78, p‐PERK, p‐IRE1α and cleaved ATF‐6 after MI/R, as well as the p‐JNK, cleaved caspase 12 and CHOP, which were directly related to the myocardial apoptosis. In vitro, decreased TAK1 activation attenuates ER stress‐mediated apoptosis induced by H/R. Thus, inhibition of TAK1 may ameliorate MI/R injury via attenuating ER stress.

MI/R injury is closely related to the activation of oxygen free radicals.^37^ Daisuke et al reported that the inhibition of p38 MAPK activity resulted in a significant decrease in the production of ROS in cardiomyocytes.^35^ Furthermore, TAK1 regulates Nrf2 through modulation of Keap‐p62/SQSTM1 interaction in the intestinal epithelium, thus affecting the production of ROS as described by Kazunori et al.^47^ Therefore, we also evaluate the role of TAK1 in oxidative stress regulation in MI/R. This study suggested that decreased TAK1 expression attenuated ROS production which was induced by MI/R. In addition, the ROS inhibitor NAC revealed the function of ROS in the process of TAK1 damage effect in H/R. The results indicated that the inhibition of ROS generation reversed the TAK1‐induced intracellular apoptosis and ER stress activation. Importantly, the ROS inhibitor NAC did not change the expression of TAK1. Moreover, TAK1 up‐regulated the expression of ER stress‐related proteins including CHOP and cleaved caspase 12, and they were partially diminished by the ROS inhibitor, indicating a potential relationship between ROS and ER stress‐induced apoptosis.

In summary, the inhibition of TAK1 activation reduces the activation of ROS and ER stress, effectively prevents myocardial apoptosis and thus ameliorates MI/R injury. The TAK1/ROS/ER stress pathway may be an essential mechanism of MI/R injury, and modulation of this signalling may be a novel strategy to prevent or interfere with this pathological process.

## CONFLICT OF INTEREST

All the authors declare that they had no conflicts of interest.

## AUTHOR CONTRIBUTIONS

Lei Li, Jingjing Zeng and Qike Jin conceived and designed the experiments. Jingjing Zeng, Qike Jin, Yongxue Ruan, Changzheng Sun and Guangyu Xu performed the experiments. Jingjing Zeng wrote the paper. Lei Li, Maoping Chu, Kangting Ji and Lianpin Wu reviewed/edited the manuscript. All authors read and approved the final version of the manuscript.

## Supporting information

Fig S1‐S3

## Data Availability

The data that support the findings of this study are available from the corresponding author upon reasonable request.
