# Supplementary material for: Inhibition of TGFβ‐activated protein kinase 1 ameliorates myocardial ischaemia/reperfusion injury via endoplasmic reticulum stress suppression
Source: J Cell Mol Med. 2020 May 7;24(12):6846–59. doi: 10.1111/jcmm.15340 (PMC7299680; doi:10.1111/jcmm.15340)
Supplement: Supplementary file 1 — Fig S1‐S3 [file JCMM-24-6846-s001.doc]

Figure 1. The mRNA level of TAK1 was not affected after MI/R injury. RT-qPCR analysising the mRNA level of TAK1 in hearts from mice subjected to sham treatment or ischemic for 0.5h followed by reperfused for oh, 1h ,2h, 4h (n=6 per time point). * Indicates p <0.05 compared with the sham group. Data are shown as means ± SEM.


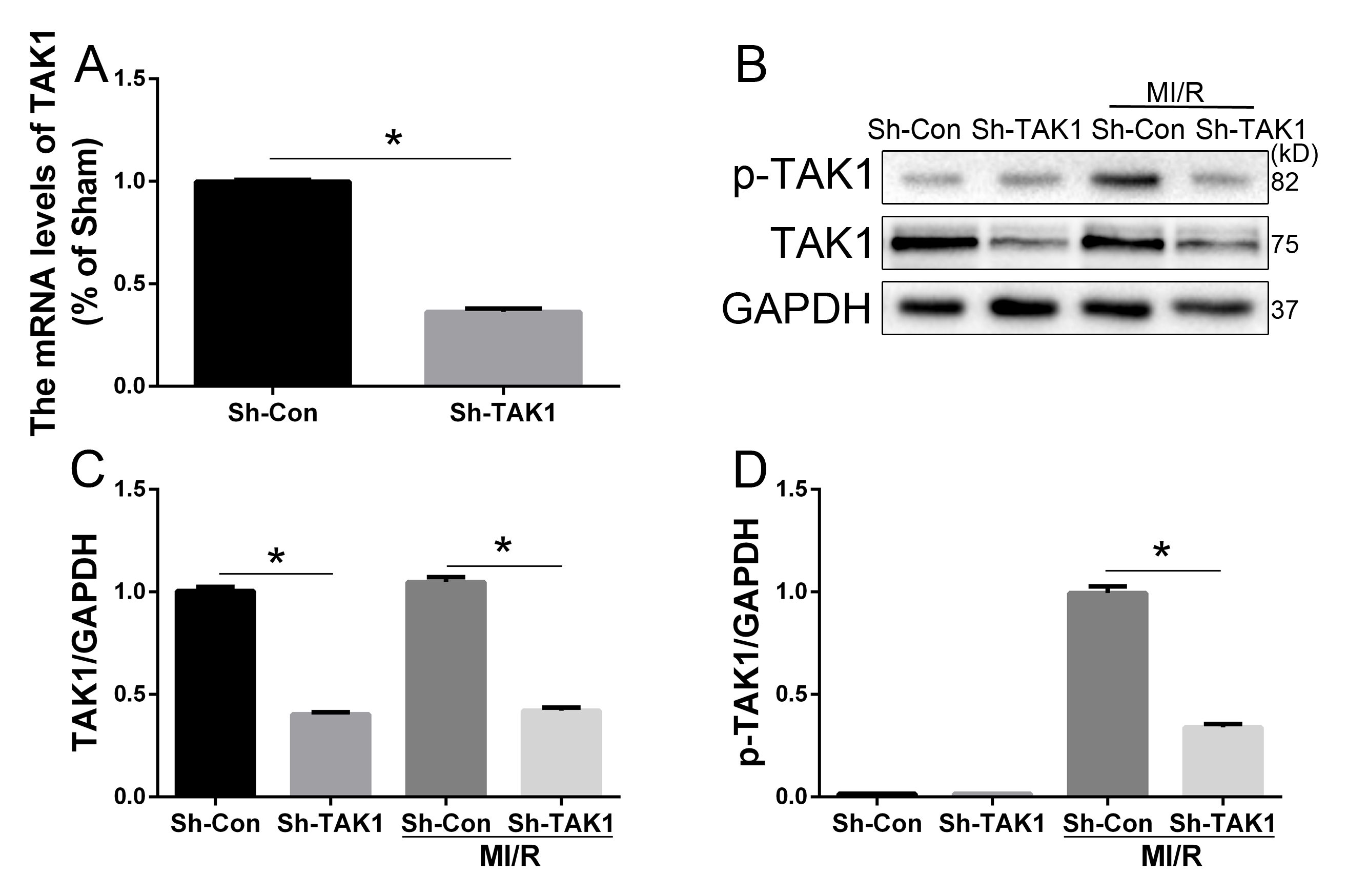


Figure 2. AAV9-cTNT-eGFP-Sh-TAK1 achieved highly efficient cardiac TAK1 silencing. (A) The mRNA level of TAK1 in the myocardium 5 weeks after AAV9 delivery. Sh-TAK1 successfully induced a nearly 70% reduction in TAK1 gene expression (n=6/group). (B) Five weeks after AAV9 vector delivery, mice were then subjected to MI/R operation. Western blot analysis of TAK1 and p-TAK1 in myocardium (n=6/group). (C) and (D) The quantitative analysis of (B). Data are shown as means ± SEM; *p < 0.05, between the indicated groups.


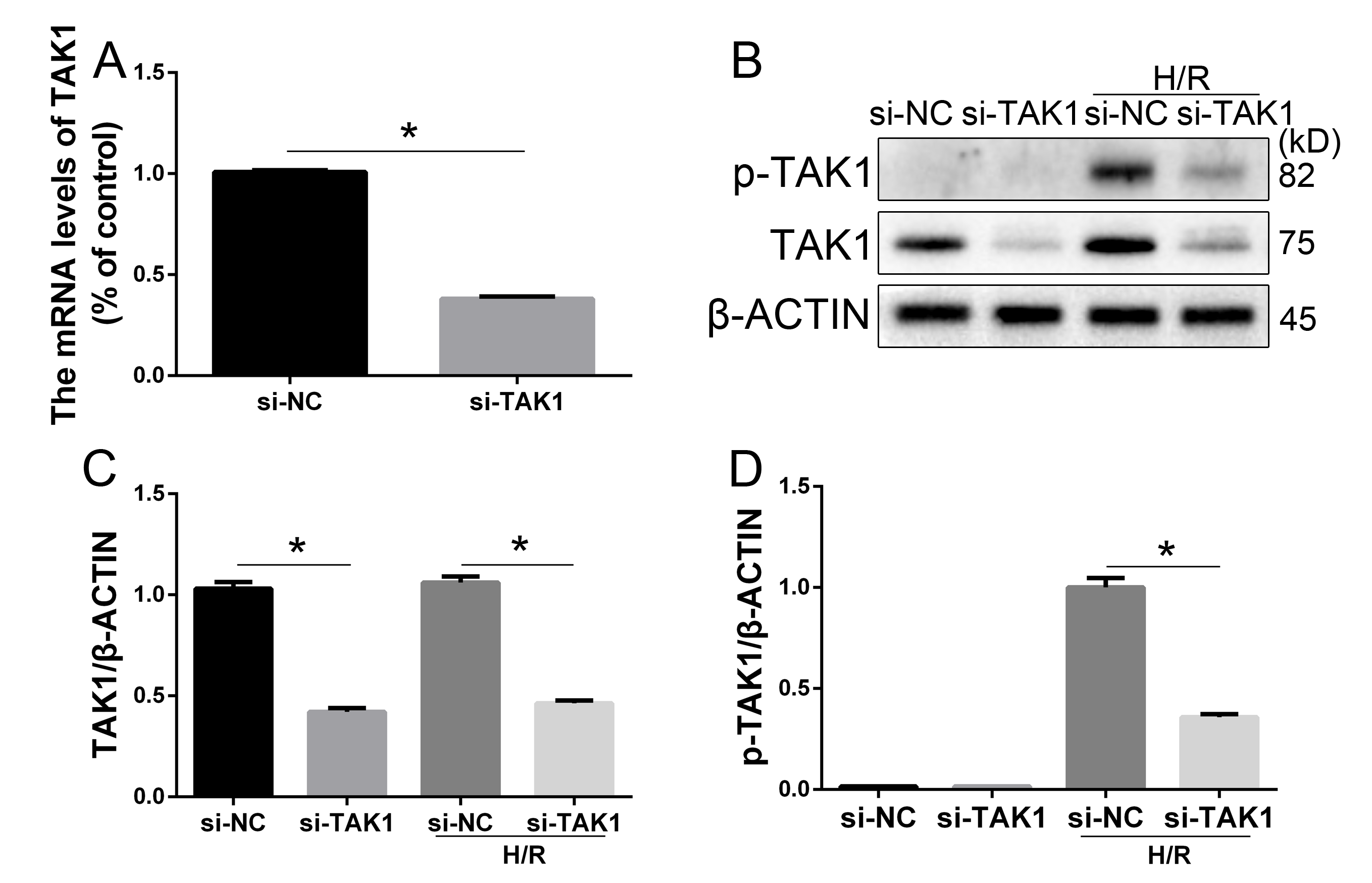


Figure 3. The effect of si-TAK1 on TAK1 silencing. (A) RT-qPCR analysis of the mRNA level of TAK1 in the cardiomyocytes (n=6/group). (B) After si-tak1 transfection, cardiomyocytes were then subjected to H/R. Western blot analysis of TAK1 and p-TAK1 in cardiomyocytes (n=6/group). (C) and (D) The quantitative analysis of (B). Data are shown as means ± SEM; *p < 0.05, between the indicated groups.
